# The role of the double layer for the pseudocapacitance of the hydrogen adsorption on platinum

**DOI:** 10.1038/s41598-022-07411-0

**Published:** 2022-03-01

**Authors:** Maximilian Schalenbach, Y. Emre Durmus, Hermann Tempel, Hans Kungl, Rüdiger-A. Eichel

**Affiliations:** Fundamental Electrochemistry (IEK‑9), Institute of Energy and Climate Research, Forschungszentrum Jülich GmbH, 52425 Jülich, Germany

**Keywords:** Electron transfer, Reaction kinetics and dynamics, Supercapacitors

## Abstract

Pseudocapacitances such as the hydrogen adsorption on platinum (HAoPt) are associated with faradaic chemical processes that appear as capacitive in their potentiodynamic response, which was reported to result from the kinetics of adsorption processes. This study discusses an alternative interpretation of the partly capacitive response of the HAoPt that is based on the proton transport of ad- or desorbed hydrogen in the double layer. Potentiodynamic perturbations of equilibrated surface states of the HAoPt lead to typical double layer responses with the characteristic resistive–capacitive relaxations that overshadow the fast adsorption kinetics. A potential-dependent double layer representation by a dynamic transmission line model incorporates the HAoPt in terms of capacitive contributions and can computationally reconstruct the charge exchanged in full range cyclic voltammetry data. The coupling of charge transfer with double layer dynamics displays a novel physicochemical theory to explain the phenomenon of pseudocapacitance and the mechanisms in thereon based supercapacitors.

## Introduction

Pseudocapacitance is a phenomenon in electrochemistry that finds applications in supercapacitors^1–4^ which are widely used as electric power buffers^5,6^. Dating back to 1941, Graham^7^ introduced the “pseudo-capacity” as a capacitive process that is based on the reversible electro-reduction of ions and that is clearly separated from the mechanism of the charge separation in the double layer. The double layer describes the ion arrangement at the electrode–electrolyte interface^8–10^. A change of the electrode potential rearranges the ions in the double layer^11–13^ and the related resistive-capacitive contributions represent part of the potentiodynamic response of every electrochemical system. In aqueous solutions, the response of the double layer to a potential variation is typically parameterized by a constant phase element^14–19^, which is a frequency domain based representation of the more complex transmission line^20^. The transmission line directly represents the potential and current distribution in the double layer and the related ion transport^11,21,22^.

In today’s science, a pseudocapacitor is understood as an electrode that stores charge indirectly through faradaic chemical processes with an electrical behavior of a capacitor^23^, covering a broad range of different types of electrochemical processes^23–25^. The hydrogen adsorption on platinum (HAoPt) appears as mainly capacitive in impedance investigations^26^. The HAoPt as a form of pseudocapacitance was discussed by Conway et al.^27^ in 1980, separating the capacitance of the double layer from that of the hydrogen adsorption. On the basis of this study, further analyses of the HAoPt were carried out using the same equivalent circuit and assumptions on the mechanisms of the pseudocapacitance^13,14,28–30^. Based on Conway’s kinetic theory on pseudocapacitance^31^, the understanding of physicochemical mechanisms of these phenomena today is that a linear dependence of the heat of adsorption on the surface coverage of the electrodeposited species leads to a potentiodynamic response in the form of a capacitance^24^. Methods to distinguish between battery-type redox reactions and pseudo capacitances are mainly based on the different shapes of these contributions to cyclic voltammetry (CV) data^32–35^.

CV is a standard method in electrochemistry that is typically operated at large amplitudes (> 0.1 V) with a triangular potential variation to characterize the transient change of surface states, electrochemical reactions and kinetics in the form of a current in the time domain^36–38^. In contrast, electrochemical impedance spectroscopy^39,40^ (EIS) typically employs small amplitudes (< 0.1 V) in the form of sinusoidal potential variations to probe perturbations of stationary states. The phase angle and amplitude of the resulting sinusoidal current is measured in the frequency domain. The intrinsic potential dependencies of electrochemical processes that arise at large amplitude probing cannot be resolved by EIS in detail^21^, however, the related impedance calculus and a simple equivalent circuit parameterization display powerful features of this method. With their different scopes and strengths, EIS and CV complement each other to probe the potentiodynamic response of electrochemical systems^21^.

The HAoPt mainly takes place at potentials below 0.4 V^41^, while each surface orientation shows an individual potential dependence in the form of a characteristic CV profile^42–44^. From a quantum mechanical point of view the HAoPt can be described with density functional theory (DFT) on an atomic scale^45–49^. Such simulations of the surface states are typically conducted for equilibrated systems, excluding the dynamics that come into play during the potentiodynamic response. Modeled CV responses of the HAoPt that are based on differences of the DFT-determined equilibrium surface coverages are reported^50,51^. Despite these approaches neglect potentiodynamic relaxation phenomena (by the double layer and the kinetic adsorption) they show in full range CV data similar transient responses of the HAoPt to those measured^41^.

The aim of this study is to present a new theory of pseudocapacitance that is based on the coupling of double layer dynamics with potentiodynamic charge transfer reactions. Hereto, EIS and CV data are collected on a polished and electro-oxidized (cycled) polycrystalline platinum sample. The potentiodynamic responses of the HAoPt to perturbations of equilibrated surface states show the typical resistive–capacitive relaxation of the double layer dynamics. EIS data at a stepwise potential variation are used to parameterize a dynamic transition line model that represents the potential dependence of the double layer. This model describes the mainly capacitive CV response at small amplitudes as well as the transient charge transfer features of the HAoPt that is examined with full range potential scans. On the basis of these results, the pseudocapacitance of the HAoPt is here discussed as a combination double layer dynamics with an instantaneous charge transfer, whereas the kinetics of the adsorption processes play a minor role in the potentiodynamic response.

## Methods

### Experimental

In this study, a freshly polished polycrystalline platinum plate in the form of a commercially-purchased sputter target (Mateck GmbH, Germany) is used as working electrode in a previously reported three-electrode cell^21^. In this in-house made cell, a geometric area of 0.79 cm^2^ of the working electrode is exposed to the electrolyte. A porous glass frit with fine pores is used to purge the electrolyte with argon, so that the amount of dissolved oxygen in the electrolyte is reduced. The platinum specimen is polished with 4000 grid SiC sandpaper and water as a lubricant. Further polishing of the sample with pastes is avoided, as these typically contain organics that contaminate the surface. Solutions of 0.01, 0.1 and 1 M HClO_4_ (Alfa Aesar) are used as the electrolytes, as perchloric acid is a superacid with an anion valence of one, displaying an ideal model electrolyte for electrochemical studies. An Ag/AgCl reference electrode (Metrohm) with a 3 M KCl electrolyte is employed, from which the potentials *vs* the reversible hydrogen electrode (RHE) are calculated by adding 0.197 V (the potential difference to the standard hydrogen electrode) and $\text{pH}\times 0.059\, \text{V}$ to compensate for the different amount of protons of the electrolytic solutions. A Metrohm Modular Line potentiostat is used for all measurements. A peak-to-peak amplitude of 0.02 V is used for all presented EIS measurements.

### Model

The measured impedance data is evaluated by an equivalent circuit consisting of a serial combination of the electrolyte resistance ${R}_{s}$ and a constant phase element (CPE) to parameterize the double layer. The CPE is characterized by a constant phase as a function of the frequency in impedance spectra. Its impedance is displayed by

1 $${Z}_{\text{CPE}}=\frac{\xi }{{\left(i\omega \right)}^{n}},$$

where $i$ denotes the complex number, $\omega$ the angular frequency, while $\xi$ and $n$ denote the parameters of the CPE. The frequency domain defined CPE can be represented by a transmission line in the form of a ladder network of resistances and capacitances^20^, which enables a time domain response modeling with differential equations^22^. Double layers typically show a potential-dependent response in impedance spectroscopy^11^. By dynamically changing the parameterization of the transmission line, the potential dependence of the double layer is implemented^22^. In comparison to a previously reported dynamic transmission model for CV data^22^, this study uses the following improvements (see Supporting Information for details and all used source codes): (i) The fits of the equivalent circuit to the impedance data were improved. (ii) The potential dependence of the CPE parameters is treated with the combination of interpolation and a Savitzky–Golay filter so that a precise description of $n$ and $\xi$ as a function of the potential is given.

## Results

The measurements presented in this study are all conducted with one polycrystalline platinum specimen without intermediate polishing. After initially polishing this specimen, EIS and CV data were recorded within a potential window between 0.05 and 0.6 V vs RHE under electrolyte variation of 0.01, 0.1 and 1 M HClO_4_. Under these conditions the surface is stable and CV cycling does not lead to a measurable change of the response. Afterwards, the same electrode was cycled between 0.05 and 1.3 V for 100 times in a 1 M HClO_4_ electrolyte, which drastically changes the CV response. Subsequently, EIS and CV measurements on the specimen were conducted again. All stated potentials refer to that of the reversible hydrogen electrode (RHE).

### EIS and CV responses to potential perturbations

**Figure 1:**
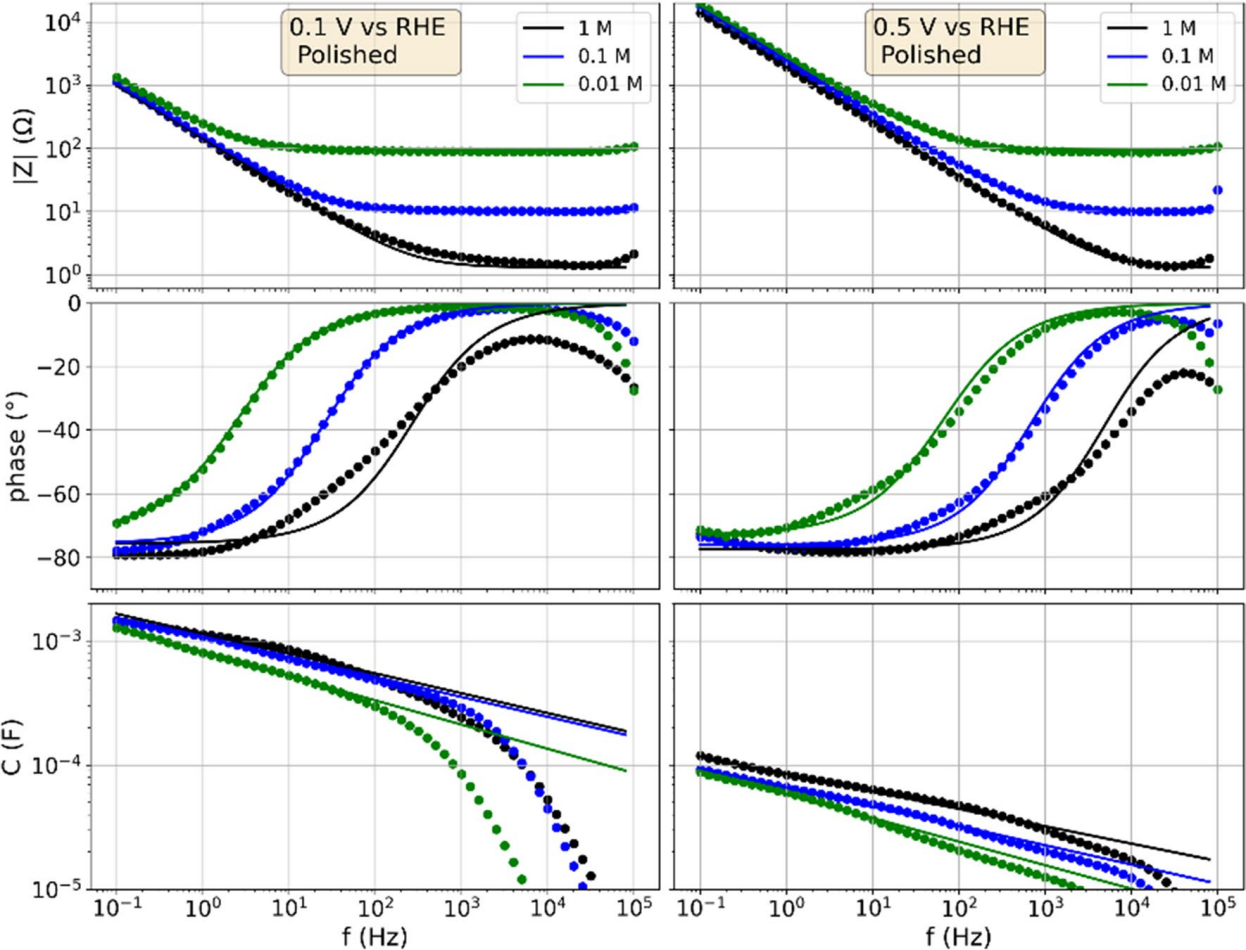
Impedance spectra and capacitance dispersion of the polished platinum specimen in 0.01, 0.1 and 1 M HClO_4_ recorded with a 20 mV peak-to-peak amplitude. Scatter: Measurements. Lines: Fits of equivalent circuit of serial resistance and constant phase element. Left column: Mean electrode potential of 0.1 V vs RHE. Right column: Mean electrode potential of 0.5 V vs RHE.

The measurements on the polished specimen presented in the following were limited to the potential range between 0.05 and 0.6 V to avoid a potential-induced surface change in the form of an oxidation. Figure 1 shows impedance spectra recorded with a peak-to-peak amplitude of 0.02 V, at a potential of 0.1 and 0.5 V using a 0.01, 0.1 and 1 M HClO_4_ electrolyte. The mean potential was applied 1 min prior to the measurements to obtain an equilibrated surface state. Besides the value of the impedance $|Z|$ and its phase angle, the capacitance dispersion is graphed, which displays the capacitive contributions to the impedance^11^. In addition, the fits of the equivalent circuit (electrolyte resistance and CPE) to the measured spectra are shown. At 0.1 V, platinum adsorbs hydrogen and the related pseudocapacitance can be observed. Between 0.4 and 0.6 V the CV response of platinum is reported as featureless, for which it is stated to be dominated by the double layer capacitance^41^.

A detailed discussion of the impedance spectra of the double layer on gold, which can be transferred to the presented data of the platinum electrode, is given elsewhere^11^. In brief, when $|Z|$ is constant, the Ohmic resistance dominates while the capacitance dispersion and the phase angle are affected by large measurement errors. The regimes of constant phase angles (here at approximately − 80°) at low frequencies are dominated by the double layer as parameterized by the CPE. The regimes between the discussed upper and lower margins are dominated by the resistive-capacitive relaxation of the double layer in combination with the electrolyte resistance.

The relaxation frequency

2 $${f}_{r}=\frac{1}{RC},$$

characterizes equal contributions of the resistive and capacitive at a phase angle of − 45° and thus is a measure for the resistive-capacitive relaxation. As reported for the double layer relaxation on the gold electrode in detail^11^, higher electrolyte concentrations decrease the resistance and with reference to Eq. (2) the relaxation frequency consequently increases. This relaxation explains the shift of the frequency dependence of the phase angle for the impedance spectra graphed in Fig. 1. Analogously, smaller capacitances increase relaxation frequency. At a frequency of 1 Hz, the spectra recorded at 0.1 V show that the capacitive contributions to the impedance are more than tenfold larger than those at 0.5 V, so that the relaxation frequency shifts by approximately the same factor.

Towards low frequencies a phase angle of − 80° is approached for all graphed impedance spectra. Excluding the regimes of constant $|Z|$ (where the measurement error dominates the capacitive contributions to the impedance), the capacitance dispersions show a constant slope in the double logarithmic plot. The variation of the electrolyte concentration by a factor of 100 induces differences of a factor of approximately 2 of the capacitance dispersions. The fits for the 0.01 and 0.1 M electrolyte show a good representation of the measured spectra. The differences between the fit and the measured data are larger at 1 M, where high currents result from low values of $\left|Z\right|$. Transport limitations related to the high currents and ion-ion interactions come into play at such high concentrations. Despite a lower precision, the fits still show a reasonable representation of the measured relaxation. The influence of electrolyte resistance and potential induced capacitance changes on the impedance spectra can be described by the theory of the double layer relaxation^11^.

**Figure 2:**
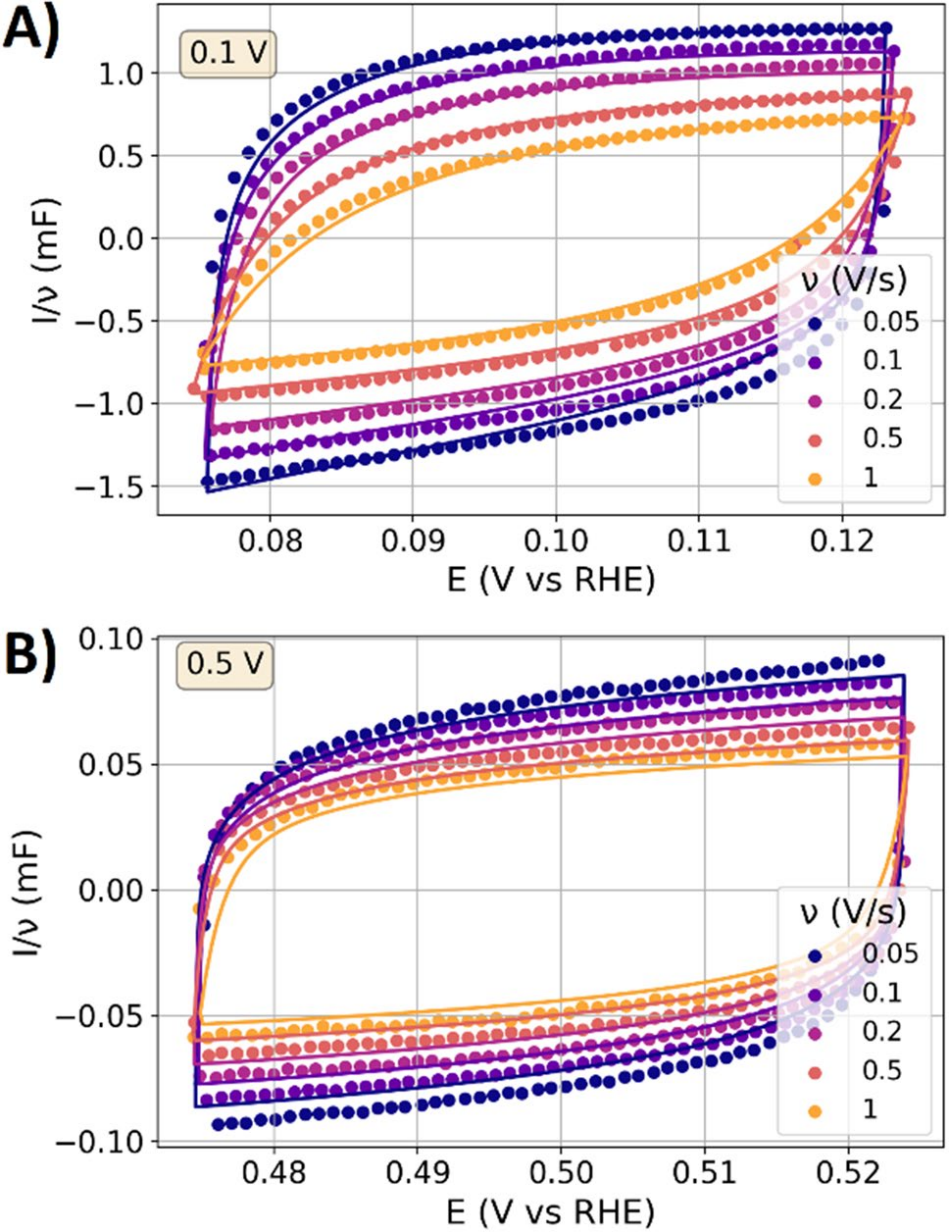
CV data with amplitudes of 0.05 V (representing a potential perturbation of the equilibrated surface state) of the polished platinum electrode with a 0.1 M HClO_4_ electrolyte. The currents are normalized to the scan rate to obtain the dimensions of a capacitance. Scatter: Measurements. Solid lines: Responses calculated with the dynamic transmission line model. (**A**) Mean potential of 0.1 V. (**B**) Mean potential of 0.5 V.

To further analyze the response in the HAoPt and double layer regime, the CV response is considered in the following, as the time domain analysis can reveal potential dependent features that are not visible in the frequency domain^21^. Hereto, the CV is examined with untypical small amplitudes of 0.05 V, which shall probe (similar to the EIS measurements) potential perturbations of equilibrated surface states. Figure 2 shows CV data of the polished sample with an electrolyte concentration of 0.1 M under a scan rate variation, again for a mean potential of 0.1 V and 0.5 V. The current is normalized to the scan rate in order to enable a comparison of the by orders of magnitude different currents that result from the scan rate variation^21^. At both mean potentials, the measured CV response displays the typical shape of the double layer response which is also observed for gold electrodes^21^. On the basis of the impedance data, the CV response is modeled with the above described dynamic transition line model, showing a good representation of the measured data.

The capacitance measured with EIS and CV is at 0.1 V more than tenfold higher than that at 0.5 V, which can be attributed to the contributions of HAoPt. At both potentials, the responses show the typical shape related to the double layer dynamics that resembles previously reported results on the double layer dynamics of a gold electrode^21^. To summarize, the perturbations of equilibrated surface states thus far indicated that the double layer dynamics dominate the responses, while the HAoPt at 0.1 V increases the measured capacitances by more than a factor of ten compared to the values at 0.5 V.

### Full range CV data

**Figure 3:**
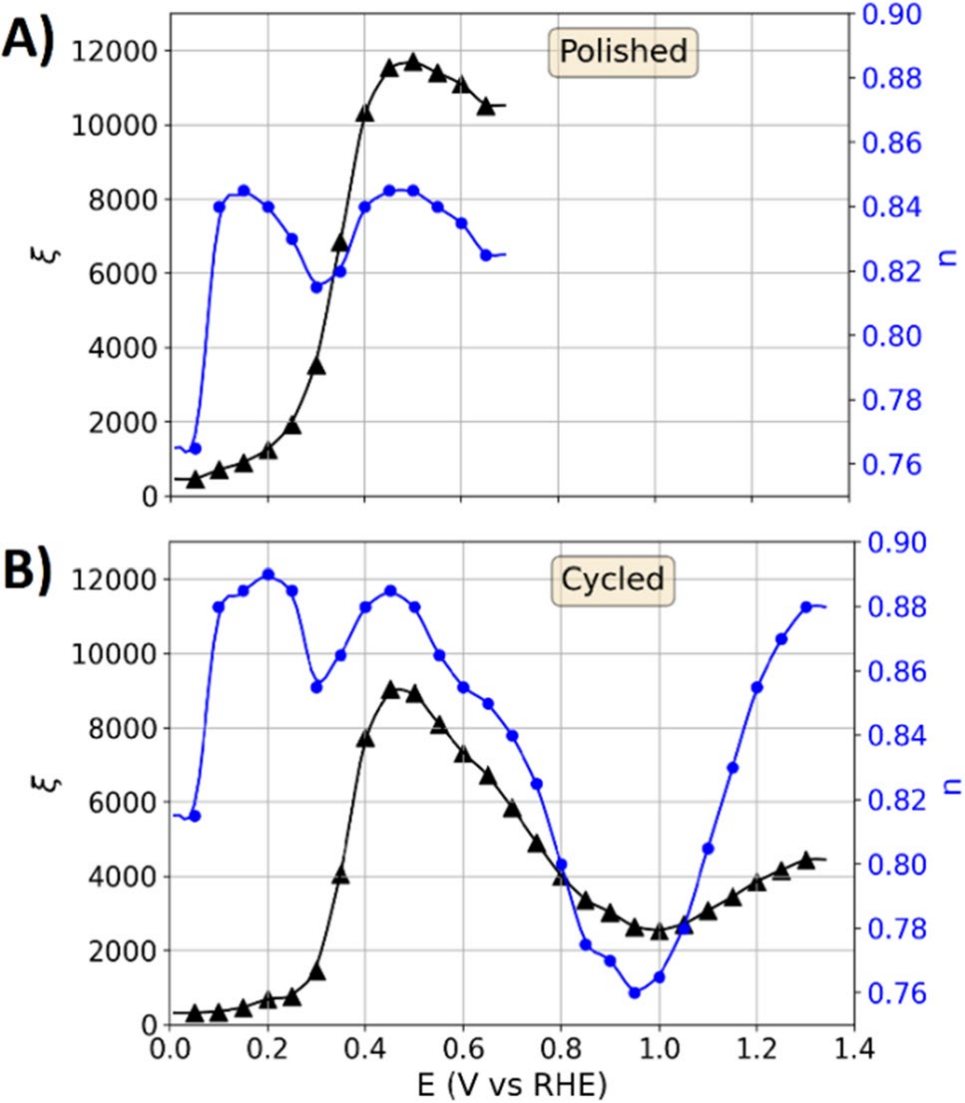
Potential dependence of the constant phase element parameterization of the double layer on the platinum specimen. Scatter: Parameters obtained from the impedance spectra at different potentials. Lines: Combination of interpolation and a Savitzky–Golay filter to achieve a continuous potential-dependent description of the parameters. (**A**) Parameterization of the polished specimen. (**B**) Parameterization of the cycled sample (with reference to Fig. 4B).

Figure 3A shows the potential dependence of the CPE parameters $\xi$ and $n$ of the polished sample as obtained by impedance measurements. After applying a potential of 0.05 V for 30 s, the first impedance spectrum was measured around this equilibrium potential with a peak-to-peak amplitude of 0.02 V. Afterwards, the potential was increased by 0.05 V and the procedure was incrementally repeated until a potential of 0.65 V was reached. To each impedance spectrum a fit is conducted in order to determine the CPE parameters $\xi$ and $n$.

**Figure 4:**
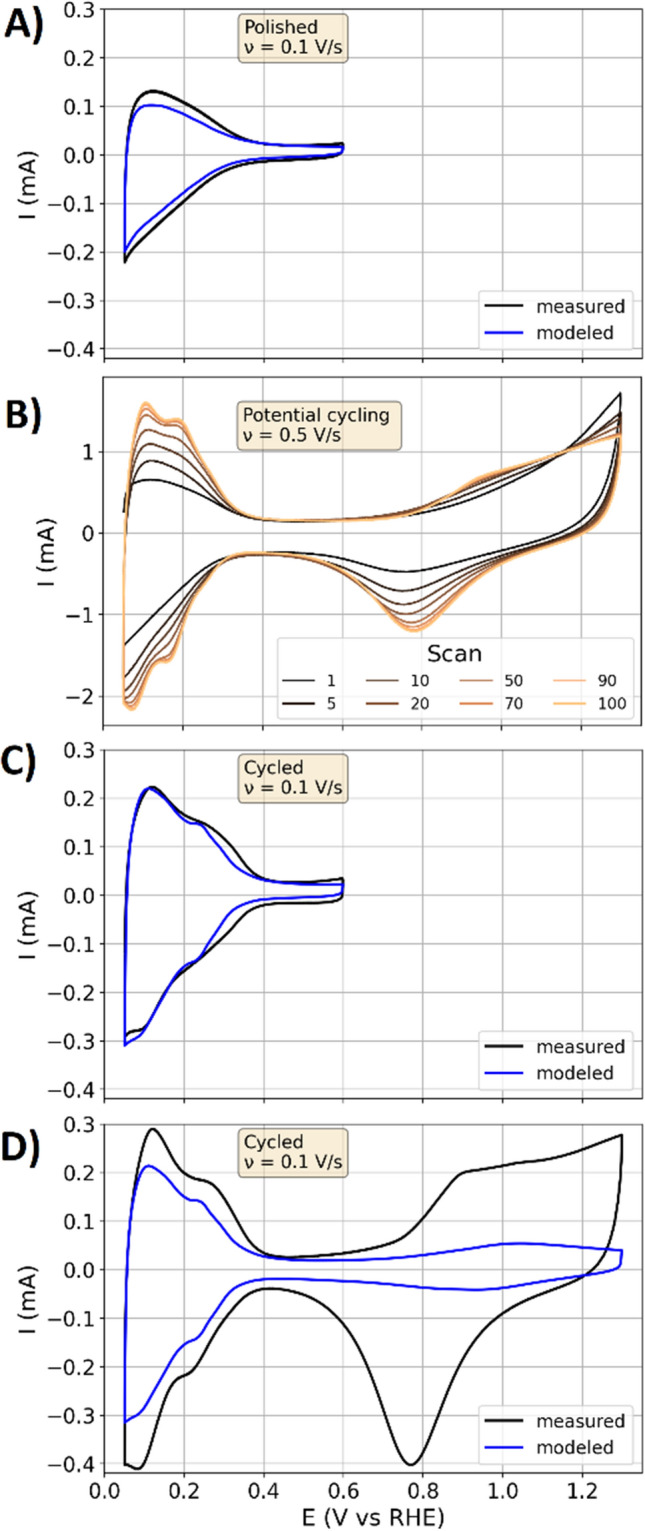
Full range CV data in chronologic order of the measurement. The modeled results were obtained with the parameterization graphed in Fig. 3 and the dynamic transmission line model. (**A**) CV data of the second scan of the polished specimen in 0.1 M HClO_4_ with a scan rate of 0.1 V/s. An upper potential limit of 0.6 V avoids oxidation. (**B**) Potential cycling of the polished sample with 100 scans in 1 M HClO_4_ and s scan rate of 0.5 V/s. (**C,D**) Measured and modeled CV data of the second scan of the cycled specimen in 0.1 M HClO_4_ with a scan rate of 0.1 V/s.

On the basis of the parameterization in Fig. 3A, the dynamic transition line model was used to calculate CV response. Figure 4A shows the thus modeled CV data and the measurements on the polished platinum specimen. The currents below 0.3 V are due to the hydrogen adsorption larger than the currents in the double layer regime between 0.4 and 0.6 V. The modeled currents are slightly smaller than the measured response, which is attributed to the amplitude dependence of the double layer parameterization (as previously reported for gold electrodes^21,22^). Large amplitudes change the ion arrangement in the double layer of the probed system and lead to asymmetric ion movement^21^, which is not probed by the small amplitude impedance parameterization presented in Fig. 3. The shape and features of the continuously changing surface states during the potential variation in the region of the hydrogen adsorption are adequately represented by the dynamic transition line model.

Figure 4B shows the change of the CV response of the polished sample during 100 cycles between 0.05 and 1.3 V. Over several decades, CV data on crystallite^52,53^, nanoparticles^54,55^ and single crystals^43,56–58^ of platinum were reported in the literature. These works excessively examined the influence of sample preparation of platinum electrodes on the measured CV response, including flame annealing and electrochemical cleaning in the form of potential cycling^41^. Full range potential cycling leads to reproducible full range CVs. However, the surface atom arrangement is changed by oxidation^59^, platinum dissolution and redeposition^60,61^, while different surface sites of single crystals come with different dissolution rates^62^. The surface atom rearrangement due to oxidation and dissolution also changes the hydrogen adsorption profile^42,63^. In agreement with these literature, the CV data changes after electro-oxidation of the polycrystalline platinum specimen with cycling to 1.3 V. Reproducible CV data with a semi-stable surface state is obtained after approximately 50 cycles. The oxidation and dissolution of the surface atoms lead to their rearrangement that results in an increase of the surface orientation with the most stable configuration^62^.

Figure 4C shows the measured CV responses with an upper potential limit of 0.6 V that were recorded after the data in Fig. 4B. Again, the modeled CV response with the dynamic transmission line model shows good agreement with the experimental data. After recording the data in Fig. 4C, the potential dependence of the double layer was parameterized as shown in Fig. 3B. Subsequently, CV responses with an upper potential limit of 1.3 V (Fig. 4D) were recorded. In comparison to Fig. 4A, additional features in the hydrogen adsorption are observed for the cycled sample, which are attributed to the changing surface orientation and structure that pronounce the adsorption on more distinct orientations of the surface sites. The CV responses graphed in Figure C and D are both modeled with the parameterization shown in Fig. 3B. However, the measurements with the upper potential limit of 1.3 V show larger currents in the range of the hydrogen adsorption than those with an upper limit of 0.6 V, showing that the semi-stable surface state after the electro-oxidation is only retained by a continuous cycling as the electrode history affects the measured response. In the case of the polished sample and the data in Fig. 4A, the surface state is stable and changes of the surface cannot be observed in CV data as long as an upper potential limit of 0.6 V is complied.

By applying larger upper potential limits than the 0.6 V, Fig. 4D also shows the potential region of the oxygen adsorption, the oxidation and reduction of the platinum surface. The modeled data in this region does not reflect the measured data. The oxygen adsorption reactions and the platinum oxidation/reduction do not appear as pseudo-capacitive contributions in the impedance spectra (see discussion in Supporting Information), for which these contributions are not included in the parameterization that is graphed in Fig. 2B. The kinetics of the reactions above 0.6 V are slower than the relaxation of the double layer, while the small peak-to-peak amplitude of 0.02 V applied during EIS does not supply enough driving force to trigger a significant change of the surface states by the sluggish reactions. Consequently, when a constant potential is applied and the surface state is equilibrated, a small perturbation of the potential does not lead to a significant contribution of the oxygen related redox-processes to the impedance spectra.

### Discussion on the pseudocapacitance of the HAoPt

The hydrogen adsorption displays a continuous variation of different adsorption states as a function of the potential^50,51^, which differs from a typical electrochemical reaction with a distinct equilibrium potential (based on the Nernst equation) that separates reducing or oxidizing conditions. Moreover, unlike a typical electrocatalytic redox-reaction where ions from the electrolyte are converted and the catalyst comes back to its initial condition, the adsorption changes the surface state of the electrocatalyst itself.

When one hydrogen atom is adsorbed at the platinum electrode, one proton is removed from the double layer. To maintain the charge balance in the double layer, a proton from the solution migrates to the double layer and is thus replacing the proton that was adsorbed. Accordingly, the hydrogen adsorption interacts with the shielding of the electrode in the double layer, for which it is directly coupled to the transport processes in the double layer. The presented data on small amplitude EIS and CV measurement at 0.1 and 0.5 V (Figs. 1, 2) showed that double layer dynamics dominated the response. As the HAoPt is a quasi-instantons process with negligible kinetic barriers a kinetic relaxation could not be observed. Thus, the ion transport mechanisms in the double layer overshadow the actual charge transfer of the adsorption process. Protons and perchloric anions can be captured in the double layer and thus both ions contribute to the transport during double layer charging/discharging. However, in the case of the hydrogen adsorption, the perchloric ions are not involved in the reaction. Nevertheless, as protons have a more than five times larger conductivity than the perchloric ions^64^ they dominate the charge transport in the double layer. As a result, the processes of double layer charging and hydrogen adsorption are both expected to be affected by similar ion transport processes in the double layer.

The resistive charge transfer is overshadowed by the ion transport in the double layer, however, it is incorporated in the form of capacitive contributions in the double layer parameterization of the dynamic transmission line model. In the case of the full range CV response, the relaxation of the double layer plays a minor role, leading to a quasi-instantaneous response to resistive-capacitive contributions of the dynamic transmission line model. Accordingly, the charge transfer of the adsorption process that is related to the differences of the surface coverage (as for instance modeled via DFT^50,51^) dominate the response. A simplified analogism can be found when a constant potential change $\Delta U$ over a capacitance $C$ leads to a constant exchanged charge $\Delta Q$ as described by:

3 $$\Delta Q=C\cdot\Delta U.$$

The dynamic transition line model includes this relation by the potential-dependent capacitive and resistive contributions of the ion transport that are described by a system of differential equations in the time domain^22^.

In the case of the gold electrode, the amplitude was reported to significantly affect the parameterization of the double layer, as the incremental parameterization by small amplitudes potential perturbations significantly underestimates the currents at high amplitudes^21^. This relation was reported to result from an asymmetric ion movement as an inert electrode displays a non-traversable border^21^. In the case of the CV response of the platinum specimen presented here, the amplitude dependence of the double layer response is less distinct as the proton adsorption decreases the asymmetry of the ion movement and related severe changes of the ion displacement in the double layer.

The reactions of the oxygen adsorption (above potentials of 0.6 V) are sluggish, so that the potential perturbations that are used in the impedance measurements just partly cause a change of the surface state (see discussion in the Supporting Information). Consequently, the related reactions just partly appear as capacitive contributions to the impedance spectra. Thus, the oxygen adsorption is not adequately parameterized by the dynamic transition line model and does not sufficiently contribute to the modeled response to resemble the experimental data in Fig. 4D. Despite the fact that the hydrogen adsorption displays the border case of an ultra-fast charge transfer whereas the oxygen adsorption represents the border case of an slow charge transfer process, at small amplitudes the double layer dominates the potentiodynamic responses in both regimes. However, the kinetic delays at the oxygen side do not allow to transfer any conclusions from the small amplitude responses to those at large amplitudes, whereas this is possible for the pseudo-capacitive hydrogen adsorption.

### Application of the new theory to other system

The HAoPt in acidic electrolyte is a kinetically fast reaction that thus displays a model system to evaluate the presented theory of pseudocapacitance, in which the capacitive contributions to the potentiodynamic response are related to the transport of ad- and desorbed ion types in the double layer. The proposed resistive-capacitive appearance of this transport process and the related relaxation is expected to display a universal phenomenon in pseudocapacitors and supercapacitors. Towards application, the charge transfer reactions in supercapacitor electrodes are typically characterized by slower kinetics than the considered HAoPt model system. It remains an open question, how these different charge transfer conditions influence the potentiodynamic response and how these interact with the ion transport in the double layer. For instance, the potentiodynamic response of the oxygen adsorption on platinum (Fig. 4D, Supporting Information) shows, that kinetically slow adsorption processes do not consequently result in pseudocapacitive contributions. The detailed transition between the regime of capacitive and kinetically delayed non-capacitive contributions to the potentiodynamic response is not well understood by now. Besides, the interaction of the adsorption energy with the surface or intercalation state that is described Conway’s kinetic theory^31^ can add to the response of the ion transport in the double layer. Detailed studies on further model electrodes and supercapacitor electrodes are necessary to understand how these different mechanisms affect the potentiodynamic response in detail. It is expected that these findings will not influence the established characterization procedures of supercapacitor electrodes, however, such insights might help in the targeted design of materials, electrodes and their power densities towards applications.

## Conclusions

This study discusses the mechanisms of the pseudo-capacitive appearance of the hydrogen adsorption on platinum (HAoPt), examined by electrochemical impedance spectroscopy (EIS) and cyclic voltammetry (CV) in combination with the computational approach of a dynamic transmission line model. First, potential perturbations of equilibrated surface states of a polycrystalline and polished platinum specimen were examined with amplitudes of 0.02 V for EIS and 0.05 V for CV. At 0.1 V, the HAoPt led to more than tenfold higher capacitances than those at 0.5 V where hydrogen and oxygen adsorption play a minor role. However, in both potential regimes, the potentiodynamic responses were dominated by the double layer with its characteristic resistive-capacitive relaxation that is described by the transmission line model. Whether ions are captured in the double layer itself or protons are adsorbed at the electrode’s surface, in both bases the ion transport must proceed through the double layer. Thereby, the double layer dynamics overshadow the kinetics of the quasi-instantaneous charge transfer of the HAoPt for which the actual charge transfer appears as pseudo-capacitive. Second, the potential-dependence of the double layer was parameterized by impedance measurements under incremental potential variation. On the basis of this parameterization, full range CV data of the platinum specimen were computationally reconstructed with the dynamic transmission line model. In contrast to the potential perturbations with small amplitudes, in the framework of the slow and large potential variations during full range CV, the transient charge transfer by the HAoPt dominates the response whereas the double layer dynamics play a minor role. By precisely describing CV responses under different amplitudes and scan rates, the dynamic transmission line model builds a bridge between the pseudo-capacitive appearance of the HAoPt at small amplitudes and its transient charge transfer character at large amplitude. Based on this detailed analysis, changes of the hydrogen surface coverages and the related charge transfer reactions are found to be inevitably connected with the ion transport in the double layer. This coupling is ultimately responsible for the dualism of the pseudo-capacitive and transient charge transfer character of the HAoPt. The discussed pseudocapacitance due to the ion transport in the double layer is expected to appear in every pseudocapacitor and supercapacitor, which (depending on the electrode material) can mix with the mechanisms described by the established kinetic theory of pseudocapacitance. To understand the combination of both mechanisms and its relation to the employed electrode materials in pseudocapacitors and supercapacitors, detailed experimental and computational studies have to follow.

## Supplementary Information

Supplementary Information.
