# Supplementary material for: The role of the double layer for the pseudocapacitance of the hydrogen adsorption on platinum
Source: Sci Rep. 2022 Mar 1;12:3375. doi: 10.1038/s41598-022-07411-0 (PMC8888654; doi:10.1038/s41598-022-07411-0)
Supplement: Supplementary file 1 — Supplementary Information. [file 41598_2022_7411_MOESM1_ESM.docx]

**Supporting information on “The role of the double layer for the pseudocapacitance of the hydrogen adsorption on platinum”**

Maximilian Schalenbach*^a^, Y. Emre Durmus ^a^, Hermann Tempel ^a^, Hans Kungl ^a^ and Rüdiger-A. Eichel ^a^

^a^ *Fundamental Electrochemistry (IEK‑9), Institute of Energy and Climate Research, Forschungszentrum Jülich GmbH, 52425 Jülich, Germany,*

*** *Corresponding author:* [*m.schalenbach@fz-juelich.de*](mailto:m.schalenbach@fz-juelich.de)

# Impedance Spectra

Figure S1 shows impedance spectra of the electro-oxidized (cycled) sample at potentials from 0.1 to 1.3 V vs RHE. The impedance spectra with the highest capacitive contributions is that at 0.1 V, which shows an at least eleven times larger capacitance than that at 0.5 V, which is dominated by double layer contributions. The spectra above 0.6 V show less than three times larger capacitive contributions to the impedance that that at 0.5 V. Thus, the increase of the capacitance in the region of the hydrogen adsorption is drastically higher than that in the oxygen adsorption.


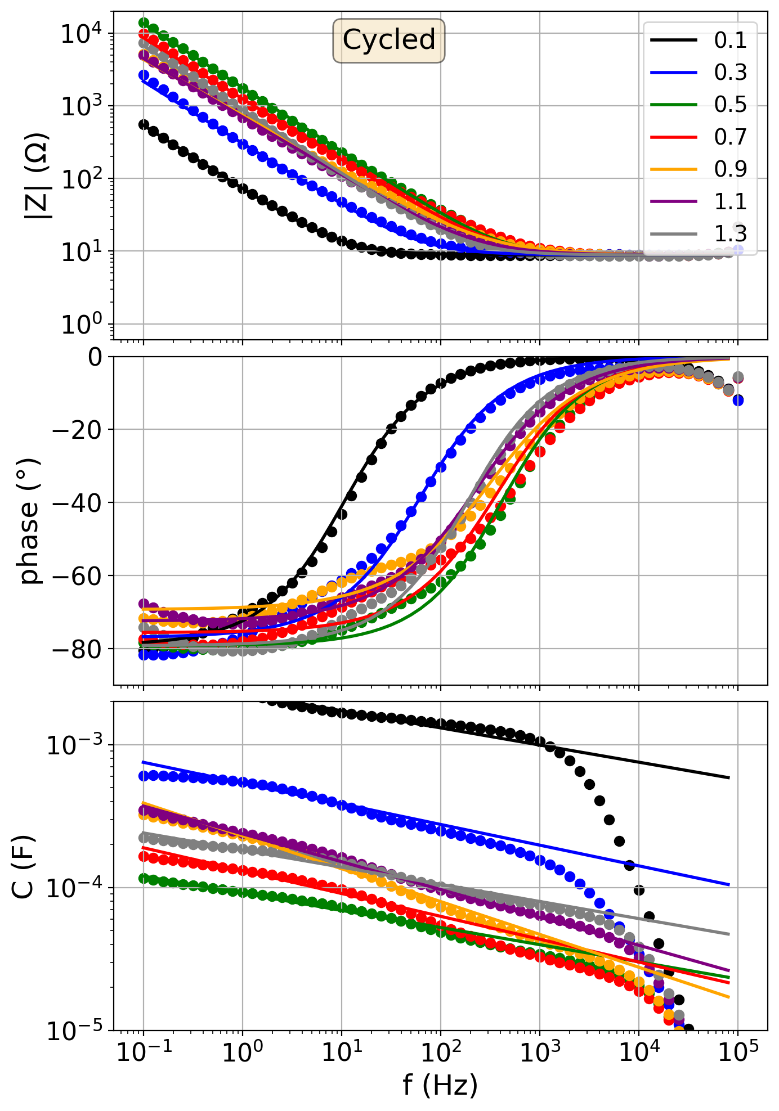


Figure S1: Impedance spectra of the cycled sample in 0.1 M HClO_4_ at different potentials between 0.1 and 1.3 V. Recorded with peak-to-peak amplitude of 0.02 V. Scatter: Measurements. Solid lines: Fits to the measured data.

In the article, Figure 4D showed that the hydrogen and oxygen adsorption lead to similar currents in the measured CV. The modeled CVs showed in the potential region of the hydrogen adsorption a good agreement with the measured data, whereas it underestimated of the currents in the potential region of the oxygen adsorption. The reason for this deviation is in the less distinct capacitive response of the oxygen evolution, which can be attributed to its sluggish kinetics.

# Source Codes

In the following, the source codes used for the data analysis and the computational model are presented. The programs are written in Python 3 and can run on personal computer without advanced hardware requirements.

## Impedance Fitting

The following code is used to fit the equivalent circuit of a serial resistance and a constant phase element to the impedance spectra

import pandas as pd

import numpy as np

import math

import matplotlib.pyplot as plt

import matplotlib

import sys, os

matplotlib.rcParams["figure.figsize"] = (7.5,5)

from scipy.optimize import curve_fit

parent_directory = os.path.dirname(os.getcwd())

sys.path.insert(0, parent_directory)

import config

Rs = config.R_s

Rct = 0# predifinition exponent of!!

pi = math.pi

n = 0 # predifinition exponent of the CPE

# =============================================================================

# Constants

# =============================================================================

lowest_frequency = config.lowest_frequency # The lowest frequency that should be ocnsidered for the fit

highest_phase_angle = config.highest_phase_angle # The highest phase angle that should be considered for the fit

#########

## Functions for 2d fit with R_ct

######

def impedance_Rs_CPE(f_array,xi):

# function to plot the fitted data

#global n

f = f_array

w = 2*math.pi*f # angular frerquency

sigma = xi*math.sin(pi*(-n)/2)*w**(-n) # substition of the real part of the CPE

rho = xi*math.cos(pi*(-n)/2)*w**(-n) # substition of the imaginary part of the CPE

Z_real = Rs + rho

Z_imag = sigma

phase = np.arctan(Z_imag/Z_real)*360/(2*pi)

cap = -1/(w*Z_imag)

Z_imag = -Z_imag

df = pd.DataFrame()

for i in ["f","cap","phase","Z_imag","Z_real"]:

df[i]= vars()[i]

df =df.set_index("f")

return df

def impedance_Rs_CPE_fit(f_array,xi):

# function for 2d fit, where Z' and Z'' are in the same vector

#global n

f = f_array[:int(len(f_array)/2)] # half of the data is redundant

w = 2*math.pi*f # angular frerquency

sigma = xi*math.sin(pi*(-n)/2)*w**(-n) # substition of the real part of the CPE

rho = xi*math.cos(pi*(-n)/2)*w**(-n)# substition of the imaginary part of the CPE

Z_real = Rs + rho

Z_imag = sigma

phase = np.arctan(Z_imag/Z_real)*360/(2*pi)

cap = -1/(w*Z_imag)

Z_imag = Z_imag

df = pd.DataFrame()

for i in ["f","cap","phase","Z_imag","Z_real"]:

df[i]= vars()[i]

df =df.set_index("f")

return np.hstack((np.log(df["Z_real"]),np.log(-df["Z_imag"]))) # return stacked Z' and Z''

def fit_main_2d_Rs_CPE(df_data_all,savepath_data_export,highest_frequency):

matplotlib.rcParams["figure.figsize"] = (7.5,5)

# =============================================================================

# remove all the data affected by measurement error

# This procedure has to be adjusted to the frequency regions of the spectrum

# that are discussed in the article

# =============================================================================

df_data_all["cap"] = -1/(2*np.pi*df_data_all["Z_imag"]*df_data_all["f"])

df_data = df_data_all[df_data_all["phase"]<highest_phase_angle]

df_data = df_data[df_data["f"]>lowest_frequency]

df_data = df_data[df_data["f"]<highest_frequency]

# =============================================================================

# Paramweterization of the measured data

# =============================================================================

# Define dataset

xdata = np.hstack((df_data["f"],df_data["f"])) # only half of it is used

ydata = np.hstack((np.log(df_data["Z_real"]),np.log(-df_data["Z_imag"])))

print(len(xdata))

# save fitparams and error

df_fiterror = pd.DataFrame(columns = ["n","popt","error"])

#fit

print(xdata)

for i in np.arange(config.n_low,config.n_high,0.005):

global n

n = i

print(n)

popt, pcov = curve_fit(impedance_Rs_CPE_fit, xdata, ydata)

df_test = impedance_Rs_CPE(df_data["f"], *popt)

plt.plot(df_test.index,df_test["phase"], label = i)

# attach errors

error = np.sum(abs(impedance_Rs_CPE_fit(xdata, *popt) - ydata))

df_fiterror.loc[i]=[n,popt,error]

del df_test

plt.xscale("log")

plt.xlabel("f (Hz)")

plt.ylabel("phase (°)")

plt.legend()

plt.scatter(df_data.f,df_data.phase)

plt.show()

#plot best fit

print("Plot best fit")

a = np.argwhere(np.asarray(df_fiterror.error) == np.asarray(df_fiterror.error).min()) # find the index of the best fit

popt = df_fiterror.iloc[a[0][0]].popt

n = df_fiterror.iloc[a[0][0]].n

xi = popt[0]

df_best = impedance_Rs_CPE(df_data_all["f"], *popt)

#plot experimental data

plt.plot(df_best.index, df_best.phase, 'r-', label='fit_data')

plt.plot(df_data_all["f"], df_data_all.phase, 'b-', label='exp_data')

plt.xscale("log")

plt.xlabel('f (Hz)')

#plt.yscale("log")

plt.ylabel('phase (°)')

plt.legend()

plt.savefig(savepath_data_export+"best_fit_phase_angle.png")

plt.show()

# plot Capacitance dispersion

print(df_fiterror.iloc[a[0][0]])

plt.plot(df_best.index, df_best.cap, 'r-', label='fit_data')

plt.plot(df_data_all["f"], df_data_all.cap, 'b-', label='exp_data')

plt.xscale("log")

plt.xlabel('f (Hz)')

plt.ylabel('C (muF)')

plt.yscale("log")

plt.legend()

plt.savefig(savepath_data_export+"best_fit_cap.png")

plt.show()

return n, xi

## Potential Dependent Parameterization

By applying the code above to impedance spectra at different potentials, the potential dependent CPE parameterization graphed in Figure 3 of the article can be obtained. To derive a continuous representation of these data, a combination of interpolation and a Savitzky–Golay filter is used by the following code:

defined_plot_margins = True

xi_max = 1.3e4

n_min = 0.75

n_max = 0.9

pot_max = 1.4

props = dict(boxstyle='round', facecolor='wheat', alpha=0.5)

import pandas as pd

pd.set_option('display.max_rows', 200)

import numpy as np

import matplotlib.pyplot as plt

import matplotlib

import os

from scipy.signal import savgol_filter

font1 = {'family' : 'normal',

'weight' : 'normal',

'size' : 18}

matplotlib.rc('font', **font1)

matplotlib.rcParams["figure.figsize"] = (8,5)

loadpath = "data"

parent_directory = os.path.dirname(os.getcwd())

loadpathroot = parent_directory + "/" + loadpath

loadpath = "data_fits_and_ladder_network"

parent_directory = os.path.dirname(os.getcwd())

def y_fit(x,params_poly_fit):

length = len(params_poly_fit.tolist())

y = np.zeros((x.shape))+params_poly_fit[length-1]

for i in range(0,length-1):

j = length-1-i

y += params_poly_fit[i]*x**j

return y

def fit(df,param,order):

#polynomial fits

params_fit_poly = np.polyfit(df.index,df[param],order,)

return params_fit_poly

# Load previously saved df

df_fit = pd.read_csv(loadpath +"/df_fit_pot_var.csv")

df_fit = df_fit.rename(columns = {"Unnamed: 0":"pot"})

df_fit["pot"] = df_fit["pot"].round(2)

df_fit = df_fit.set_index("pot")

df_fit = df_fit.sort_index()

x = np.arange(df_fit.index.min()-0.05,df_fit.index.max()+0.05,0.001)

df_fit2 = df_fit.copy() # introducing some interpolation at the margins, so that polynomial fitting works better

# df_fit2 = df_fit2[df_fit2.index < 0.6]

min_index = df_fit2.index.min()

max_index = df_fit2.index.max()

for j in df_fit2.index:

if j < df_fit2.index.max():

for i in [0.04,0.03,0.02,0.01]:

df_fit2.loc[j+i] = [np.nan,np.nan, np.nan]

df_fit2 = df_fit2.sort_index()

df_fit2 = df_fit2.interpolate()

# Fill end with last numbers

for i in [0.04,0.03,0.02,0.01]:

df_fit2.loc[min_index-i] = df_fit2.loc[min_index]

df_fit2.loc[max_index+i] = df_fit2.loc[max_index]

df_fit2 = df_fit2.sort_index()

print(df_fit2)

df_fit2["xi_smoothed"] = savgol_filter(df_fit2.xi,11,4)

df_fit2["n_smoothed"] = savgol_filter(df_fit2.n,11,4)

df_fit2.to_csv("df_params_smoothed.csv")

df_fit2.to_csv(parent_directory+"/codes_continous/df_params_smoothed.csv")

# =============================================================================

# plot CPE params

# =============================================================================

color_tuples = []

len_spectra = len(df_fit)

for i in range(0,len_spectra): color_tuples.append(tuple([1-i/len_spectra,0,i/len_spectra]))

fig, ax1 = plt.subplots()

arrow_l = int(len(df_fit)/4)

arrow_r = int(len(df_fit)*3/4)

color = 'tab:red'

ax1.set_xlabel('E (V vs RHE)')

ax1.set_ylabel(r'$\xi$', color='k')

#ax1.plot(df_fit.index, df_fit.xi, 'k:')

#ax1.plot(x,y_fit(x,params_fit_poly_xi),'k:')

ax1.tick_params(axis='y', labelcolor='k')

ax1.plot(df_fit2.index, df_fit2["xi_smoothed"],"k")

for i in range(len(df_fit.index)):

ax1.scatter(list(df_fit.index)[i],df_fit.at[list(df_fit.index)[i],"xi"],marker = "^",s=100,color = "k")#color_tuples[i])

y_spread = abs(df_fit.xi.max()-df_fit.xi.min())

ax2 = ax1.twinx() # instantiate a second axes that shares the same x-axis

ax2.set_ylabel('n', color='b') # we already handled the x-label with ax1

ax2.plot(df_fit2.index, df_fit2.n_smoothed,"b")

ax2.tick_params(axis='y', labelcolor='b')

for i in range(len(df_fit.index)):

ax2.scatter(list(df_fit.index)[i],df_fit.at[list(df_fit.index)[i],"n"],color = "b")#color_tuples[i])

y_spread = abs(df_fit.n.max()-df_fit.n.min())

#ax2.arrow(list(df_fit.index)[arrow_r],df_fit.at[list(df_fit.index)[arrow_r],"n"],0.1,0,width= y_spread/100,head_width = y_spread/25,head_length =0.02,color="k")

fig.tight_layout() # otherwise the right y-label is slightly clipped

if defined_plot_margins:

plt.xlim(0,pot_max)

ax1.set_ylim(0,xi_max)

ax2.set_ylim(n_min,n_max)

ax1.grid()

ax1.text(0.8, 0.95*xi_max, "Polished", fontsize=18, verticalalignment='top', bbox=props)

plt.savefig(loadpath + "/fit_params.png", dpi = 200)

plt.show()

## Transmission Line Model

Using the parameterization of the code above, the dynamic transmission line model can be used to calculate the cyclic voltammetry response.

import sys

import os

import shutil

import pandas as pd

import numpy as np

import matplotlib.pyplot as plt

import math

import scipy.special as scs

import matplotlib

matplotlib.rc('xtick', labelsize=16)

matplotlib.rc('ytick', labelsize=16)

font1 = {'family' : 'normal',

'weight' : 'normal',

'size' : 18}

matplotlib.rc('font', **font1)

matplotlib.rcParams["figure.figsize"] = (7.5,5)

parent_directory = os.path.dirname(os.getcwd())

sys.path.insert(0, parent_directory)

import config

import fit_poly

# =============================================================================

# #### Start: Main parameters ####

# =============================================================================

# Model option

CV_continous = True # False: only one set of n & xi, True: Potetnial depednent n & xi

voltage_range_from_experiment = True

experimental_correction = True

# Model parameters

ladder = 50 # Ladder length

h_resolution = 0.001

# CV and EQC params

scan_rate = 0.1

Rs = config.R_s # electrolyte resistance

C_DE = config.C_DE

Rct = config.C_DE

# static parameters (only if no exp is loaded)

n_static = 0.875

xi_static = 57208

U_start_CV = 0.5

amp = 0.05

df_params_smoothed = pd.read_csv("df_params_smoothed.csv")

df_cv_table = pd.read_csv(parent_directory+"/tables/cv_table.csv")

for i in [14]:#np.arange(1,2,1):#[1,10,17,24]:#[0,1,2,3,4,5,7,8,9,10]:

scan_rate = df_cv_table.at[i, "scan_rate"]

print("Scan rate : "+str(round(scan_rate,3)))

print("Measurement number : "+str(i))

measurement_number = i

unique_foldername = "CV_"+str(measurement_number)+config.EQC # the foldder name where to save stuff

# =============================================================================

# Main functions and code excecution

# =============================================================================

# Parameteritazation for continous ladder calculation

if voltage_range_from_experiment == True:

if measurement_number != 0:

path_CV = parent_directory + "/data/cv("+str(measurement_number)+").txt"

else:

path_CV = parent_directory + "/data/cv.txt"

df_CV = pd.read_csv(path_CV, sep = "\t")

dict_rename = {'WE(1).Potential (V)':"U",'WE(1).Current (A)':"I" }

df_CV = df_CV.rename(columns = dict_rename)

df_CV["U"] = df_CV["U"] + 0.197+config.pH*0.059

exp_min_max = fit_poly.return_min_max_CV(df_CV)

U_start_CV = exp_min_max[0]

amp = exp_min_max[1]-exp_min_max[0]

if CV_continous == True:

# do CV fitting and parameter calulation

params_poly_fit_CV1_up, params_poly_fit_CV1_down, params_poly_fit_CV2_up, params_poly_fit_CV2_down = fit_poly.fit_CV(df_CV)

ladder_vec = np.arange(0,ladder,1)

def calc_ln_vec(h,ladder,n,xi):

"""

wie calc_ln nur als Vektor

"""

ln = np.zeros((3,ladder))

def P(n):

return math.gamma(1-n)/math.gamma(n)

P_n = P(n)

ln[0,:] = ladder_vec.copy()

#calculate resistances

ln[1,ladder_vec] = 2*(h**n)*P_n*scs.gamma(ladder_vec+n)/scs.gamma(ladder_vec+1-n)

ln[1,0] = 2*h**n*P_n*math.gamma(n)/math.gamma(1-n) - h**n

#calculate capacitances

ln[2,ladder_vec] = h**(1-n)*(2*ladder_vec+1)*scs.gamma(ladder_vec+1-n)/(P_n*scs.gamma(ladder_vec+1+n))

ln[1,:] = xi*ln[1,:]

ln[2,:] = ln[2,:]/xi

return ln

def CPE_num(U_start, amp, scan_rate,experimental_correction):

# Create directory

saveroot = os.getcwd() + "/results_" + config.EQC

if os.path.exists(saveroot)==False:

os.mkdir(saveroot)

savepath = saveroot +"/" + unique_foldername

print(savepath)

# make new folder with the experimental Settings

if os.path.exists(savepath):

shutil.rmtree(savepath)

os.mkdir(savepath)

print('scan rate '+str(scan_rate))

# ladder calculation

if CV_continous == False: #if the ladder should be calculated only once

dummy, R, C = calc_ln_vec(h_resolution,ladder,n_static,xi_static)

# =============================================================================

# Time descritization

# =============================================================================

t_total = amp/scan_rate # time for 1/2 of triangular function

# g = step szize

g = 0.00002 #Time intervall (s)

tend = int(t_total/g) #Time intervall (s)

print("steps : "+str(tend))

# =============================================================================

# Numerical caluclation

# =============================================================================

print("Numerically calculating the response")

#ladder+1 = total

I = np.zeros((4*tend+1,ladder))*0.0001

IR = np.zeros((4*tend+1,ladder))*0.0001

IR_Rs = np.zeros((4*tend+1))*0.0001

IR_Rct = np.zeros((4*tend+1))*0.0001

It = np.zeros((4*tend+1))*0.0001

time = np.zeros((4*tend+1))

U = np.zeros((4*tend+1)) + U_start

I_diff = np.zeros((4*tend+1))

Scan_array = np.zeros((4*tend+1))

print("starting the numerical calculation of the ladder network response")

for period in [0,1,2,3]:

if period%2 ==0 : # even periods are up-sweeps

nu = scan_rate

down = 0

else: # odd periods are down-sweeps

nu = -scan_rate

down = 1

print(str(period)+" sweep")

# time iteration and resistive current calculation

for j in range(0,tend):

j0 = j

j = period*tend + j

# Parmaeters of up- or down scan

time[j+1] = period*t_total + g*(j0+1)

U[j+1] = U_start + down*amp + nu*g*(j0+1)

# recalculate ladder network

if CV_continous == True:

n = np.interp(U[j+1],df_params_smoothed.pot,df_params_smoothed.n_smoothed)

xi = np.interp(U[j+1],df_params_smoothed.pot,df_params_smoothed.xi_smoothed)

dummy, R, C = calc_ln_vec(h_resolution,ladder,n,xi)

#R[0] += Rs

### last step of the ladder network

m = ladder-1

IR[j+1,m] = 1/R[m]*((IR[j,m-1]-IR[j,m])/C[m-1]-(IR[j,m])/C[m])*g + IR[j,m]

# non-boundary steps of the ladder network

for k in range(1, ladder-1):

m = ladder-1-k

IR[j+1,m] = 1/R[m]*((IR[j,m-1]-IR[j,m])/C[m-1]-(IR[j,m]-IR[j,m+1])/C[m])*g + IR[j,m]

IR[j+1,0] = 1/(R[0]+Rs)*(nu - (IR[j,0]-IR[j,1])/C[0])*g + IR[j,0]

IR_Rs[j+1] = IR[j+1,0] # both currents are the same!!

# =============================================================================

# Collect data

# =============================================================================

#It = IC_tot

It = IR_Rs

I = IR # total current through CPE

df = pd.DataFrame()

df["t"] = time

df["U"] = U

df["I"] = It

df["I_diff"] = I_diff

Scan_array[0*tend:1*tend-1] = 0

Scan_array[1*tend:2*tend-1] = 1

Scan_array[2*tend:3*tend-1] = 2

Scan_array[3*tend:4*tend-1] = 3

df["Scan_array"] = Scan_array

df.iloc[::20, :].to_csv(savepath+"/df.csv", index=False)

df2 = df.copy()

for i in range(0,ladder-1):

df2["R"+str(i)] = IR[:,i]

df2.iloc[::20, :].to_csv(savepath+"/df2.csv", index=False)

# =============================================================================

# # Plotting

# =============================================================================

#plt.title("response in time domain")

fig, ax1 = plt.subplots()

ax2 = ax1.twinx()

ax1.plot(time[0:4*tend-1],U[0:4*tend-1],'k')

ax2.plot(time[0:4*tend-1],It[0:4*tend-1]*1e6,'b')

ax1.set_xlabel("time (s)")

ax1.set_ylabel('U (V)', color='k')

ax2.set_ylabel('I (µA)', color='b')

ax2.tick_params(axis='y', colors='blue')

plt.savefig(savepath+"/response.png")

plt.show()

#plt.title("voltage-current characteristic")

plt.plot(U[0*tend:2*tend-1],It[0*tend:2*tend-1]*1e6,'k:')

plt.plot(U[2*tend:4*tend-1],It[2*tend:4*tend-1]*1e6,'k')

#plt.plot(U[0:4tend],I[0:4tend,0]*1e6/nu*ladder,'r')

#plt.plot(U[0:tend],I[0:tend,10]*1e6/nu*ladder,'g')

plt.xlabel("U (V)")

plt.ylabel("I (µA)")

plt.plot(df_CV.U, df_CV.I*1e6, "b")

plt.savefig(savepath+"/modeled_and_measured.png",dpi=200)

plt.show()

#plt.title("Difference current")

#plt.plot(U[0*tend:2*tend-1],I_diff[0*tend:2*tend-1]*1e6,'k:')

plt.plot(U[2*tend+100:3*tend-100],I_diff[2*tend+100:3*tend-100]*1e6,'k:')

plt.plot(U[3*tend+100:4*tend-100],I_diff[3*tend+100:4*tend-100]*1e6,'k')

plt.xlabel("U (V)")

plt.ylabel("I$\mathregular{_{diff}}$ (µA)")

plt.show()

#plt.title("maximimal current of each ladder step")

x = []

y = []

for i in range(0,ladder):

x.append(i)

y.append(I[3*tend:4*tend-1,i].max()*1e6)

max_I_ladder = np.asarray(y).round(6)

plt.plot(x,y)

plt.yscale('log')

plt.ylabel('I$\mathregular{_{max}}$ (µA)')

plt.xlabel("ladder step")

plt.savefig(savepath+"/max_current.png",dpi=200)

plt.show()

cmap = plt.get_cmap("copper",ladder)

#plt.title("normalized current of each ladder step")

for i in range(0,ladder):

if i==0:

label = "k = 0"

elif i==ladder-1:

label = "k = "+str(i)

else:

label = '_nolegend_'

plt.plot(time[2*tend:4*tend-1],I[2*tend:4*tend-1,i]/I[2*tend:4*tend-1,i].max(), color = cmap(i),label = label)

plt.ylabel('normlaized current (a.u.)')

plt.xlabel("time (s)")

#plt.legend()

plt.savefig(savepath+"/normalized_currents.png",dpi=200)

plt.show()

#return [h,ladder,nu, max_I_ladder]

# Code excecution

experimental_correction = False

CPE_num(U_start_CV, amp, scan_rate,experimental_correction)
